# Unique continuation for the magnetic Schrödinger equation

**DOI:** 10.1002/qua.26149

**Published:** 2020-01-25

**Authors:** Andre Laestadius, Michael Benedicks, Markus Penz

**Affiliations:** ^1^ Department of Chemistry, Hylleraas Centre for Quantum Molecular Sciences University of Oslo Oslo Norway; ^2^ Department of Mathematics Uppsala University Uppsala Sweden; ^3^ Max Planck Institute for the Structure and Dynamics of Matter Hamburg Germany

**Keywords:** Hohenberg‐Kohn theorem, Kato class, magnetic Schrödinger equation, molecular Hamiltonian, unique‐continuation property

## Abstract

The unique‐continuation property from sets of positive measure is here proven for the many‐body magnetic Schrödinger equation. This property guarantees that if a solution of the Schrödinger equation vanishes on a set of positive measure, then it is identically zero. We explicitly consider potentials written as sums of either one‐body or two‐body functions, typical for Hamiltonians in many‐body quantum mechanics. As a special case, we are able to treat atomic and molecular Hamiltonians. The unique‐continuation property plays an important role in density‐functional theories, which underpins its relevance in quantum chemistry.

## Graphical abstract

The unique‐continuation property from sets of positive measure for the Schrödinger equation states that a solution cannot vanish unless it is equal to zero everywhere. This property is an important ingredient in the proof of the Hohenberg‐Kohn theorem, which is a fundamental result in quantum chemistry stating that the one‐body particle density determines the external potential. This work addresses this property and its relevance in current density functional theory that extends density functional theory to include systems with magnetic fields.

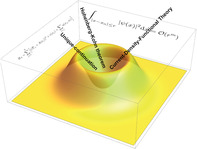

## 1 INTRODUCTION

Within the Schrödinger model for quantum systems of (interacting) electrons, in order to be able to describe interesting phenomena like the Zeeman effect, the quantum Hall effect, or the Hofstadter butterfly one has to include the effects of both an electric and a magnetic field. Hohenberg and Kohn showed for systems without magnetic fields that the one‐body ground‐state particle density determines the electric (scalar) potential up to a constant.^1^ Strictly speaking, the particle density determines at most one potential (modulo an additive constant) since some densities are not associated with any potential.^2^ The above correspondence between densities and potentials constitutes the theoretical foundation on which density functional theory (DFT)—a ubiquitous tool in quantum chemistry and materials science^3^, ^4^ —is built.

In the presence of magnetic fields though, the approach of Hohenberg and Kohn to set up a universal density functional requires more than just the particle density due to the fact that an additional vector potential enters the system's Hamiltonian. Both the paramagnetic current density and the total (physical) current density have been suggested as basic variables alongside the particle density^5^, ^6^, ^7^ and the resulting framework is called current density functional theory (CDFT). For the theory that uses the paramagnetic current density, counterexamples to a Hohenberg‐Kohn theorem are known,^8^, ^9^ although a weaker version still holds. Note that even for degenerate systems the weaker version is enough to define a universal paramagnetic current density functional.^10^ Diener has presented an argument^7^ for establishing a full Hohenberg‐Kohn theorem using the total current density. However, as first noted in Reference 11, the argument is at best incomplete.

For more detailed accounts on the existence of generalized Hohenberg‐Kohn theorems within CDFT see References 9, 11, 12, and for related and positive results within the Maxwell‐Schrödinger theory and quantum‐electrodynamical DFT see References 13, 14, 15. An interesting and recent development is also given in Reference 16 where the existence of generalized Hohenberg‐Kohn theorems is further explored. A different route, where a Hohenberg‐Kohn result comes for free by virtue of the convex‐analytic properties of a *regularized* energy functional was taken in References 17, 18. It was specifically implemented for CDFT in Reference 19 and can even be used to prove convergence of the associated Kohn‐Sham iteration Scheme.^20^

The current work arises as a natural ingredient for proving a generalized Hohenberg‐Kohn theorem in total (physical) CDFT. It addresses the property that a solution of the magnetic Schrödinger equation cannot vanish on a set of positive measure, a property called unique continuation, see Definition 1. Unique continuation is also a fundamental property for solutions of the magnetic Schrödinger equation in its own right and has been well‐studied.^21^, ^22^, ^23^, ^24^, ^25^, ^26^ In the context of CDFT the issue was first raised in Reference 9. As far as DFT and CDFT are concerned, it is useful to have the assumptions guaranteeing the unique‐continuation property as particle‐number independent as possible (at least avoid increasing integrability constraints with increasing *N*), which is a difficult task. In the present work we obtain results that are adapted to the many‐body Schrödinger equation and that furthermore include vector potentials, building on the results of Kurata^25^ and Regbaoui.^26^ This means that the specific structure of the potentials is beneficially taken into account. The main results, Theorem 6 and Corollary 8, that include the singular Coulomb potentials of atoms and molecules as a special case (Corollary 9), are formulated in terms of the Kato class $K_{\mathrm{loc}}^{n}$ and its generalization $K_{\mathrm{loc}}^{n,\delta }$, with *n* = 3 and *n* = 6 (Definition 3). Although we cannot answer the question of the existence of a generalized Hohenberg‐Kohn theorem for the total current in CDFT, we exemplify the use of the unique‐continuation property in a limited special case (Corollary 11).

## 2 UNIQUE‐CONTINUATION PROPERTY AND THE HOHENBERG‐KOHN THEOREM

In the most simple setting of only one particle and without vector potential, it is known that the (unique) ground state *ψ* in *H* ^1^(ℝ^3^) can be chosen to be strictly positive, see Theorem 11.8 in Lieb‐Loss.^27^ This means that we can set *ρ* ^1/2^ = *ψ* > 0 and the following relation to the scalar potential *v* must hold (from the Schrödinger equation, here written as [−Δ + *v*]*ψ* = *eψ*)

(1) $$v\left(x\right)=e+\frac{\Delta \rho {\left(x\right)}^{1/2}}{\rho {\left(x\right)}^{1/2}},\;x\;\in \;{\mathbb{R}}^{3},$$

where Δ denotes the Laplacian and *e* is the ground‐state energy. Conversely, given a particle density *ρ* we can ask if a potential *v* exists such that the given *ρ* is the ground‐state density of that potential. For the one‐particle case, this problem has been studied by Englisch and Englisch^2^ and was answered in the negative even for well‐behaved densities (*N*‐representable densities). Corollary 3 in Reference 28 (including the additional constraint Δ*ρ* ^1/2^ ≤ *Cρ* ^1/2^ and $\rho^{-1}\;\in \;L_{\mathrm{loc}}^{1}$ besides *N*‐representability) provides sufficient conditions for one‐particle *v*‐representability, that is, *v* can be computed from *ρ* as given in Equation (1) and *ρ* is the ground‐state density of that *v*.

Returning to the general *N*‐electron case without magnetic field, we first recall the Hohenberg‐Kohn theorem: Given two systems, if *ρ*_1_ = *ρ*_2_ then *v*_1_ = *v*_2_ + constant, where *ρ*_*k*_, *k* = 1,2, is the ground‐state particle density of the corresponding system defined by the potential *v*_*k*_. The proof of this result relies on the fact that if *ψ* is a ground state of both systems, then $\sum_{k=1}^{N}\left(v_{1}\left(x_{k}\right)-v_{2}\left(x_{k}\right)\right)\psi =\text{constant}\times \psi$. If *ψ* does not vanish on a set of positive (Lebesgue) measure we have *v*_1_ = *v*_2_+ constant almost everywhere. The proof can then be completed by means of the variational principle, using the Hohenberg‐Kohn argument by reductio ad absurdum.^1^ (Note that a strict inequality in the variational principle is not needed, see, eg, Reference 29 and that the results also hold for systems with degeneracy.^2^)

In this article we address the more general case of *N* interacting, nonrelativistic (spinless) particles subjected to both a scalar and a vector potential. The fundamental question then is, whether any eigenfunction of the corresponding Hamiltonian

(2) $$H_{N}=\sum_{j=1}^{N}\left[{\left(i\nabla_{j}+A\left(x_{j}\right)\right)}^{2}+v\left(x_{j}\right)+\sum_{l<j}u\left(x_{j},x_{l}\right)\right]$$

can be zero on a set of positive measure without being identically zero. This is a problem of *unique continuation*.

Definition 1 We say that the Schrödinger equation H_N_ψ = eψ has the unique‐continuation property (UCP) from sets of positive (Lebesgue) measure if a solution that satisfies ψ = 0 on a set of positive measure is identically zero. Furthermore, the Schrödinger equation is said to have the strong UCP if whenever ψ vanishes to infinite order at some point x_0_, that is, for all m > 0

$$\int_{|x-x_{0}|\leq r}{\left|\psi \left(x\right)\right|}^{2}dx=O\left(r^{m}\right)\;\left(r\to 0\right),$$

then ψ is identically zero. Additionally, if ψ = 0 on a non‐empty open set implies that ψ is identically zero, then the Schrödinger equation has the weak UCP.

Remark 1 The strong UCP implies the weak UCP. The UCP from sets of positive measure allows us to conclude ψ ≠ 0 almost everywhere for any eigenfunction of H_N_.

There exists a considerable amount of literature that treats the UCP for differential inequality |Δ*ψ*| ≤ |*ξ*_1_||∇*ψ*| + |*ξ*_2_||*ψ*|.^21^, ^22^, ^23^, ^24^, ^25^, ^26^ In particular if $\xi_{1}\;\in \;L_{\mathrm{loc}}^{n}\left({\mathbb{R}}^{n}\right)$ and $\xi_{2}\;\in \;L_{\mathrm{loc}}^{n/2}\left({\mathbb{R}}^{n}\right)$, the corresponding differential equation Δ*ψ* = *ξ*_1_ ·∇*ψ* + *ξ*_2_ *ψ* has the UCP from sets of positive measure.^26^ Note that such $L_{\mathrm{loc}}^{p}$ constraints become more restrictive with increasing particle number, since the dimension of the configuration space *n* enters in the conditions. Directly applied to *H*_*N*_ *ψ* = *eψ* this means that if a solution *ψ* in the Sobolev space $H_{\mathrm{loc}}^{2N/\left(N+2\right)}\left({\mathbb{R}}^{3N}\right)$ vanishes on a set of positive measure,

$$\begin{array}{c} \sum_{j=1}^{N}[v\left(x_{j}\right)+{\left|A\left(x_{j}\right)\right|}^{2}+i\left(\nabla_{j}\cdot A\left(x_{j}\right)\right)+\sum_{l<j}u\left(x_{j},x_{l}\right)]\;\in \;L_{\mathrm{loc}}^{3N/2}\left({\mathbb{R}}^{3N}\right), \end{array}$$

and each component of *A* belongs to $L_{\mathrm{loc}}^{3N}\left({\mathbb{R}}^{3N}\right)$, then *ψ* is identically zero.

Such results are used by Lammert,^30^ particularly in his Theorem 5.1, to give a mathematically precise proof of the Hohenberg‐Kohn theorem^1^ in DFT including the UCP as remarked by Lieb.^31^ Yet he does not consider magnetic fields and the constraints are very susceptible to the particle number. A recent effort by Garrigue^29^ removed the dependence on particle numbers for the constraints on the scalar potential by exploiting their specific shape in the context of many‐body (molecular) Hamiltonians. Reference 29 also contains a rigorous proof of the Hohenberg‐Kohn theorem including all the mathematical details for potentials $v\;\in \;L_{\mathrm{loc}}^{p}\left({\mathbb{R}}^{3}\right)$, *p* > 2.

## 3 PREREQUISITES

Let the Hamiltonian *H*_*N*_ be as in Equation (2). The Schrödinger equation is then given by *H*_*N*_ *ψ* = *eψ*. We write *H*_*N*_ = *T*_*A*_ + *V* + *U*, where $T_{A}=\sum_{j=1}^{N}{\left(i\nabla_{j}+A\left(x_{j}\right)\right)}^{2},\;\nabla_{j}=\left(\frac{\partial }{\partial x_{j}^{1}},\frac{\partial }{\partial x_{j}^{2}},\frac{\partial }{\partial x_{j}^{3}}\right)$ and $x_{j}=\left(x_{j}^{1},x_{j}^{2},x_{j}^{3}\right)\;\in \;{\mathbb{R}}^{3}$ are the coordinates of the *j*th electron. Here we use (*i*∇_*j*_ + *A*(*x*_*j*_))^2^ in *T*_*A*_ instead of (−*i*∇_*j*_ + *A*(*x*_*j*_))^2^ in order to follow the notation in Kurata.^25^ We use a slight variation of atomic units *ℏ* = 2*m*_*e*_ = 1 and *q*_*e*_ = −1, such that the Laplace operator appears without a factor 1/2.

The electric potential *V* is a one‐body potential given by $V\left(x\right)=\sum_{j=1}^{N}v\left(x_{j}\right)$ with *v* : ℝ^3^ → ℝ. The two‐particle interaction *U* between the electrons is modeled by *U*(*x*) = ∑_1 ≤ *j* < *l* ≤ *N*_ *u*(*x*_*j*_, *x*_*l*_), for some nonnegative function *u* on ℝ^3^ × ℝ^3^ . We set *W* = *V* + *U*. Furthermore, *A* : ℝ^3^ → ℝ^3^ denotes the vector potential, from which the magnetic field is obtained by *B* = ∇ × *A*. With the notation $\underline{A}\left(x\right)={\left(A\left(x_{j}\right)\right)}_{j=1}^{N}$, the Schrödinger equation is rewritten as

(3) $$-\Delta \psi +2i\underline{A}\cdot \nabla \psi +\left(W_{A}-e\right)\psi =0,$$

where $W_{A}=W+{\left|\underline{A}\right|}^{2}+i\left(\nabla \cdot \underline{A}\right)$.

A function $f\;\in \;L_{\mathrm{loc}}^{2}\left({\mathbb{R}}^{n}\right)$ belongs to the Sobolev space $H_{\mathrm{loc}}^{k}\left({\mathbb{R}}^{n}\right)$ if *f* has weak derivatives up to order *k* that belong to $L_{\mathrm{loc}}^{2}\left({\mathbb{R}}^{n}\right)$. Let the set of infinitely differentiable functions with compact support on ℝ^3*N*^ be denoted by $C_{0}^{\infty }\left({\mathbb{R}}^{3N}\right)$. We say that $\psi \;\in \;H_{\mathrm{loc}}^{1}\left({\mathbb{R}}^{3N}\right)$ is a solution of Equation (3) in the weak sense, which will be our standard notion for solutions from here on, if for all $\varphi \;\in \;C_{0}^{\infty }\left({\mathbb{R}}^{3N}\right)$

(4) $$\begin{array}{c} \int_{{\mathbb{R}}^{3N}}\nabla \psi \cdot \nabla \bar{\varphi }\;dx+2i\int_{{\mathbb{R}}^{3N}}\underline{A}\cdot \left(\nabla \psi \right)\bar{\varphi }\;dx+\int_{{\mathbb{R}}^{3N}}\left(W_{A}-e\right)\psi \bar{\varphi }\;dx=0. \end{array}$$

The present work takes off from the following result:

Theorem 2 (Theorem 1.2 in Regbaoui^26^). *Let* N ≥ *1. Assume that* $W_{A}\;\in \;L_{\mathrm{loc}}^{3N/2}\left({\mathbb{R}}^{3N}\right)$ *and each component of* A *is an element of* $L_{\mathrm{loc}}^{3N}\left({\mathbb{R}}^{3N}\right)$. *Then the Schrödinger equation has the UCP from sets of positive measure, that is, if a solution* $\psi \;\in \;H_{\mathrm{loc}}^{2N/\left(N+2\right)}\left({\mathbb{R}}^{3N}\right)$ *vanishes on a set of positive measure then it is identically zero*.

Remark 2 See also Theorem 1.1 in Regbaoui^26^ for the strong UCP and Wolff^23^ for the weak UCP.

If one employs Theorem 2 with *N* = 1, since there is no two‐particle interaction it suffices to assume that *v,|A|* ^2^ and ∇· *A* are elements of $L_{\mathrm{loc}}^{3/2}\left({\mathbb{R}}^{3}\right)$ to obtain the UCP from sets of positive measure. With increasing particle number, however, the assumptions on the potentials *v*, *u*, and *A* are such that they rule out most types of singularities. On the other hand, the particle‐number dependence that enters in $H_{\mathrm{loc}}^{2N/\left(N+2\right)}$ fulfills the inequality 2 *N*/(*N* + 2) ≤ 2 for all *N*. Following Kurata^25^ the $L_{\mathrm{loc}}^{p}$ constraints, with *p* proportional to *N*, can be avoided.

Definition 3 A function $f\;\in \;L_{\mathrm{loc}}^{1}\left({\mathbb{R}}^{n}\right)$ belongs to the Kato class $K_{\mathrm{loc}}^{n}$, n ≠ 2, if for every R > 0, $\lim_{r\to 0+}\eta^{K}\left(r;f\right)=0$, where

$$\eta^{K}\left(r;f\right)=\underset{|x|\leq R}{\sup}\int_{B_{r}\left(x\right)}\frac{|f\left(y\right)|}{{\left|x-y\right|}^{n-2}}dy.$$

Furthermore, $f\;\in \;K_{\mathrm{loc}}^{n,\delta }\subset K_{\mathrm{loc}}^{n}$, δ > 0, if for every R > 0

$$\lim_{r\to 0+}\underset{|x|\leq R}{\sup}\int_{B_{r}\left(x\right)}\frac{|f\left(y\right)|}{{\left|x-y\right|}^{n-2+\delta }}dy=0.$$

We write *f* = *f*_+_ − *f*_−_, where *f*_−_ (*f*_+_) is the negative (positive) part of *f* given by *f*_−_(*x*) = max(−*f*(*x*), 0) ( *f*_+_(*x*) = max(*f*(*x*), 0)). Let *y* ∈ ℝ^3*N*^ be fixed and for *x* = (*x*_1_, …, *x*_*N*_) ∈ ℝ^3*N*^ (cf. the notation in Kurata^25^)

$$\begin{array}{l} a\left(x\right)={\left|\underline{A}\left(x\right)\right|}^{2},\\ b_{y}\left(x\right)={\left|x-y\right|}^{2}\sum_{j=1}^{N}{\left|B\left(x_{j}\right)\right|}^{2},\\ Q_{y}\left(x\right)={\left(2W+\left(x-y\right)\cdot \nabla W\right)}_{-}\;. \end{array}$$

With the notation above, we formulate.

Assumption 1 1 Suppose

$$\begin{array}{l} \nabla \cdot A\;\in \;L_{\mathrm{loc}}^{2}\left({\mathbb{R}}^{3}\right),\\ A^{j}\;\in \;L_{\mathrm{loc}}^{4}\left({\mathbb{R}}^{3}\right)\;\left(A^{j}\text{component of}\;A\right),\\ a,b_{y},W,Q_{y}\;\in \;K_{\mathrm{loc}}^{3N}\;\left(\text{for fixed}\;y\;\in \;{\mathbb{R}}^{3N}\right), \end{array}$$

and that for some *r*_0_ > 0

(5) $$\int_{0}^{r_{0}}\frac{\theta_{y}\left(r\right)}{r}dr<\infty$$

holds, where θ_*y*_(r) = η^K^(r; Q_y_) + η^K^(r; b_y_)^1/2^ .

Remark 3 Remark 1.2 in Kurata^25^ gives $b_{y},Q_{y}\;\in \;K_{\mathrm{loc}}^{3N,\delta }$, for some *δ* > 0, as a sufficient condition for Equation (5) to hold.

Remark 4 The main condition in *Assumption* 1 is with respect to the Kato class $K_{\mathrm{loc}}^{n}$, n = 3 N being the dimensionality of the underlying configuration space. This condition is *optimal* in the sense that the class cannot be enlarged to *smaller* orders than n = 3 N, or the UCP will be lost. This follows from the inclusion $L_{\mathrm{loc}}^{p}\subset K_{\mathrm{loc}}^{n}$ for all p > n/2 and a sharp counterexample provided in Reference 32 for a potential in L^p^, p < n/2. So if the order of the Kato class would be any m < n then it also includes $L_{\mathrm{loc}}^{p}$ with m/2 < p < n/2 and that is ruled out by the given counterexample.

The following is obtained by adapting Corollary 1.1 in Kurata^25^ (denoted Lemma 5 below). For the sake of simplicity, and since it is enough for our purposes here, we make the restrictions to real‐valued *V* and *U*. In the sequel we use the notation |*F*| for the Frobenius norm (also called the Hilbert‐Schmidt norm) of a matrix (*F*_*j*,*l*_)_*j*,*l*_.

Theorem 4 *Suppose Assumption* 1 *. If* $\psi \;\in \;H_{\mathrm{loc}}^{2}\left({\mathbb{R}}^{3N}\right)$ *is a solution of (3) and vanishes to infinite order at* x_0_ ∈ *R* ^3N^ , *then* ψ *is identically zero. Thus the Schrödinger equation has the strong UCP*.

Lemma 1 (Corollary 1.1 in Kurata^25^). *Let* n ≥ *3*, x_0_ ∈ ℝ^*n*^ *be fixed*, $x=\left(x^{1},\ldots ,x^{n}\right)\;\in \;{\mathbb{R}}^{n},\;\tilde{A}=\left({\tilde{A}}^{1},\ldots ,{\tilde{A}}^{n}\right):{\mathbb{R}}^{n}\to {\mathbb{R}}^{n},\;\tilde{W}:{\mathbb{R}}^{n}\to \mathbb{R}$, $F={\left(F_{j,l}\right)}_{j,l=1}^{n}$ *with* $F_{j,l}=\partial {\tilde{A}}^{j}/\partial x^{l}-\partial {\tilde{A}}^{l}/\partial x^{j}$, *and suppose that*

(6) $$\begin{array}{l} {\tilde{A}}^{j}\;\in \;L_{\mathrm{loc}}^{4}\left({\mathbb{R}}^{n}\right),\;\nabla \cdot \tilde{A}\;\in \;L_{\mathrm{loc}}^{2}\left({\mathbb{R}}^{n}\right),\;{\left|\tilde{A}\right|}^{2}\;\in \;K_{\mathrm{loc}}^{n},\\ {\left(\left|x-x_{0}\right|\left|F\right|\right)}^{2}\;\in \;K_{\mathrm{loc}}^{n}, \end{array}$$

*and*

(7) $$\tilde{W}\;\in \;K_{\mathrm{loc}}^{n},\;{\left(2\tilde{W}+\left(x-x_{0}\right)\cdot \nabla \tilde{W}\right)}_{-}\;\in \;K_{\mathrm{loc}}^{n}.$$

Furthermore assume, for some *r*_0_ > 0,

(8) $$\begin{array}{l} \int_{0}^{r_{0}}[\eta^{K}\left(r;{\left(2\tilde{W}+\left(x-x_{0}\right)\cdot \nabla \tilde{W}\right)}_{-}\right)\\ +\eta^{K}{\left(r;{\left(|x-x_{0}|\;|F|\right)}^{2}\right)}^{1/2}]\frac{dr}{r}<\infty \end{array}$$

holds. Then if $\psi \;\in \;H_{\mathrm{loc}}^{2}\left({\mathbb{R}}^{n}\right)$ satisfies

(9) $$\left(\sum_{j=1}^{n}{\left(i\frac{\partial }{\partial x^{j}}+{\tilde{A}}^{j}\left(x\right)\right)}^{2}+\tilde{W}\left(x\right)\right)\psi =0$$

and vanishes to infinite order at *x*_0_, it follows that *ψ* is identically zero.

Proof of Theorem 4 *We will show that Assumption 1 directly fulfills all the conditions of Lemma 5 that thus becomes applicable. Let* n = 3 N and $\tilde{W}$ = W − *e*. *By Assumption 1, (7) is then fulfilled. The choice* Ã = A *implies* $T_{A}={\left(i\nabla +\tilde{A}\right)}^{2}$ *and Equation (3) can be written as Equation (9)*.

*Each component of* $A\;\in \;L_{\mathrm{loc}}^{4}\left({\mathbb{R}}^{3}\right)$ *yields* ${\tilde{A}}^{j}\;\in \;L_{\mathrm{loc}}^{4}\left({\mathbb{R}}^{3N}\right)$ *for j* = 1, …, 3 N. *From* $\nabla \cdot \tilde{A}=\sum_{k=1}^{N}\nabla_{k}\cdot A\left(x_{k}\right)$ *and* $\nabla \cdot A\;\in \;L_{\mathrm{loc}}^{2}\left({\mathbb{R}}^{3}\right)$, *we obtain* $\nabla \cdot \tilde{A}\;\in \;L_{\mathrm{loc}}^{2}\left({\mathbb{R}}^{3N}\right)$. *Since a* = |*Ã*|^2^, *it holds that* ${\left|\tilde{A}\right|}^{2}\;\in \;K_{\mathrm{loc}}^{3N}$. *Moreover, the matrix* F *satisfies*

(10) $$\begin{array}{l} {\left(\left|x-x_{0}\right|\left|F\right|\right)}^{2}={\left|x-x_{0}\right|}^{2}\sum_{j,l=1}^{3N}{\left|F_{j,l}\right|}^{2}=2b_{x_{0}}\left(x\right), \end{array}$$

*since *F *contains* N *repeated blocks of sub matrices of the form*

$$\left[\begin{array}{ccc} 0 & -B_{3}\left(x_{j}\right) & B_{2}\left(x_{j}\right)\\ B_{3}\left(x_{j}\right) & 0 & -B_{1}\left(x_{j}\right)\\ -B_{2}\left(x_{j}\right) & B_{1}\left(x_{j}\right) & 0 \end{array}\right].$$

This establishes Equation (6).

From Equation (5), $\tilde{W}$ = *W* − *e* and Equation (10), we conclude that Equation (8) holds. Lemma 5 gives the strong UCP for Equation (3) and the proof is complete. □

Remark 5 As stated in Remark 1.1 in Kurata,^25^ Lemma 5 and thus Theorem 4 also holds if in *Assumption* 1, $K_{\mathrm{loc}}^{3N}$ is replaced by $K_{\mathrm{loc}}^{3N}+F_{\mathrm{loc}}^{p}\left({\mathbb{R}}^{3N}\right)$, 1 < p ≤ 3 N/2. Here $F_{\mathrm{loc}}^{p}\left({\mathbb{R}}^{3N}\right)$ is the Fefferman‐Phong class and in this case a solution must be an element of $H_{\mathrm{loc}}^{2}\left({\mathbb{R}}^{3N}\right)\cap L_{\mathrm{loc}}^{\infty }\left({\mathbb{R}}^{3N}\right)$, and there is an additional condition on *V*_−_.

## 4 MAIN RESULTS

Theorem 4 above establishes the strong UCP under Assumption 1. If in addition the negative part of *v* is locally *L* ^3/2^(ℝ^3^) summable we obtain the UCP from sets of positive measure:

Theorem 6 *Suppose Assumption 1* 1 *. If in addition* $v_{-}\;\in \;L_{\mathrm{loc}}^{3/2}\left({\mathbb{R}}^{3}\right)$ *and* $\psi \;\in \;H_{\mathrm{loc}}^{2}\left({\mathbb{R}}^{3N}\right)$ *solves* 3 *and vanishes on a set of positive measure, then* ψ *is identically zero. Consequently, the Schrödinger equation has the UCP from sets of positive measure*.

Remark 6 The requirement u ≥ 0 can be relaxed if one assumes that u(*x*_1_, *x*_2_) = u^*′*^(*x*_1_ − *x*_2_) (see Lemma A.2 in Lammert^30^).

If the strong UCP can be obtained under other assumptions than Assumption 1, the following corollary can be used to obtain the UCP from sets of positive measure.

Corollary 7 *Suppose the strong UCP for the Schrödinger equation (not necessarily by means of Assumption* 1), *then the constraint* $v_{-}+{\left|A\right|}^{2}+i\left(\nabla \cdot A\right)\;\in \;L_{\mathrm{loc}}^{3/2}\left({\mathbb{R}}^{3}\right)$ *gives the UCP from sets of positive measure*.

Due to the particular form of the potentials, we can write

$$W\left(x\right)=\sum_{j}v\left(x_{j}\right)+\frac{1}{2}\sum_{j\neq l}u\left(x_{j},x_{l}\right).$$

Because *Q*_*y*_ is defined as the negative part of the function 2*W* + (*x* − *y*)·∇*W*, we have with the choice ${\underline{x}}_{0}=\left(x_{0},\ldots ,x_{0}\right)\;\in \;{\mathbb{R}}^{3N}$, for fixed *x*_0_ ∈ ℝ^3^ ,

(11) $$0\leq Q_{x_{0}}\left(x\right)=Q_{{\underline{x}}_{0}}\left(x\right)\leq \sum_{j}q_{1;x_{0}}\left(x_{j}\right)+\sum_{j\neq l}q_{2;x_{0}}\left(x_{j},x_{l}\right)$$

for some functions $q_{1;x_{0}}$ and $q_{2;x_{0}}$. (See below the proof of Corollary 3, where this decomposition is done for the choice of *W* corresponding to the molecular case.) Furthermore,

$$b_{x_{0}}\left(x\right)=b_{{\underline{x}}_{0}}\left(x\right)=\sum_{j,l=1}^{N}{\left|x_{j}-x_{0}\right|}^{2}{\left|B\left(x_{l}\right)\right|}^{2}$$

can be split as

$$b_{x_{0}}\left(x\right)=\sum_{j}b_{1;x_{0}}\left(x_{j}\right)+\sum_{j\neq l}b_{2;x_{0}}\left(x_{j},x_{l}\right).$$

We can now formulate our main result that includes *H_N_* modeling atoms and molecules, and where the exponents in the integrability constraints are independent of the particle number *N*.

Corollary 8 *For* N ≥ *2 and* x_0_ ∈ ℝ^3^ *fixed, suppose*

(12) $$\begin{array}{cc} & \nabla \cdot A\;\in \;L_{\mathrm{loc}}^{2}\left({\mathbb{R}}^{3}\right),\;A^{j}\;\in \;L_{\mathrm{loc}}^{4}\left({\mathbb{R}}^{3}\right),\;{\left|A\right|}^{2}\;\in \;K_{\mathrm{loc}}^{3},\\ & b_{1;x_{0}}\;\in \;K_{\mathrm{loc}}^{3,\delta },\;b_{2;x_{0}}\;\in \;K_{\mathrm{loc}}^{6,\delta }. \end{array}$$

*Further, let* $v_{-}\;\in \;L_{\mathrm{loc}}^{3/2}\left({\mathbb{R}}^{3}\right),\;v\;\in \;K_{\mathrm{loc}}^{3}$ *, and* $u\;\in \;K_{\mathrm{loc}}^{6}$ *, as well as* $Q_{x_{0}}$ *satisfying* 11 *with* $q_{1;x_{0}}\;\in \;K_{\mathrm{loc}}^{3,\delta }$ *and* $q_{2;x_{0}}\;\in \;K_{\mathrm{loc}}^{6,\delta }$ *. Then the Schrödinger equation* 3 *has the UCP from sets of positive measure*.

In particular, the magnetic Schrödinger equation has the UCP from sets of positive measure for *H_N_* modeling atoms and molecules in magnetic fields if just Equation (12) is fulfilled.

Corollary 9 *For* N ≥ *2, suppose the magnetic field is such that Equation* 12 *holds. Then with*

$$v\left(x_{1}\right)=-\sum_{j=1}^{M_{\mathrm{nuc}}}\frac{Z_{j}}{|x_{\mathrm{nuc};j}-x_{1}|},\;u\left(x_{1},x_{2}\right)=\frac{1}{|x_{1}-x_{2}|},$$

*where* x_nuc;j_ ∈ ℝ^3^ *and* Z_j_ > *0 are the positions and charges of the* M_nuc_ *nuclei, respectively, the UCP from sets of positive measure holds for the Schrödinger equation*.

## 5 APPLICATION TO CDFT

In the presence of a magnetic field, no equivalence of a (general) Hohenberg‐Kohn result exists at present.^9^, ^11^ However, we shall now address how the UCP from sets of positive measure for the magnetic Schrödinger equation plays an important role in the argument for restricted Hohenberg‐Kohn theorems in CDFT and the nonuniversal variant magnetic‐field density‐functional theory (BDFT) of Grayce and Harris.^33^ Given a wave function *ψ*, define the particle density and the paramagnetic current density according to

ρψx=N∫ℝ3N−1ψ(xx2…xN)2dx2…dxN,jψpx=NIm∫ℝ3N−1ψ¯xx2…xN∇xψxx2…xNdx2…dxN.

For a vector potential *A* we may compute the total current density by the sum $j=j_{\psi }^{p}+\rho_{\psi }A$. Now, fix the particle number *N* as well as the two‐particle interaction *u* (eg, *u*(*x*_1_, *x*_2_) = |*x*_1_ − *x*_2_|^−1^ ) and write *H*_*N*_ = *H*(*v*,*A*). If *ψ* is a ground state for some *v* and *A*, that is, *H*(*v*,*A*)*ψ* = *eψ*, where *e* is the ground‐state energy, then *ρ*_ψ_, $j_{\psi }^{p}$, and $j=j_{\psi }^{p}+\rho_{\psi }A$ are called ground‐state densities of *H*(*v*,*A*). Whether the ground‐state particle density *ρ* and the total current density *j* determine *v* and *A* (up to a gauge transformation) is still an open question in the general case.^9^, ^11^ (For the ground‐state density pair (*ρ*,*j* ^*p*^) it is well‐known that this density pair does not determine the potentials *v* and *A*.^8^)

Now, assume that two systems with Hamiltonians *H*(*v*_1_,*A*_1_) and *H*(*v*_2_,*A*_2_) have the same ground‐state particle density (i.e., *ρ*_1_ = *ρ*_2_ = *ρ*) and ∇ × *A*_1_ = ∇ × *A*_2_ = *B*. Suppose that *v*_*k*_, *A*_*k*_ for *k* = 1,2, and *B* fulfill Assumption 1 1 and the requirements given in Theorem 6. Since there exists a function *f* such that *A*_1_ = *A*_2_ − ∇*f*, the variational principle yields

$$e_{1}\leq 〈\psi_{2},H\left(v_{1},A_{1}\right)\psi_{2}〉\leq e_{2}+\int_{{\mathbb{R}}^{2}}\left(v_{1}-v_{2}\right)\rho dx,$$

where *ψ*_2_ is the ground state of *H*(*v*_2_, *A*_2_ − ∇*f*). Switching the indices, we find that

$$e_{1}-e_{2}=\int_{{\mathbb{R}}^{2}}\left(v_{1}-v_{2}\right)\rho dx.$$

Consequently, *ψ*_2_ is a ground state of both *H*(*v*_1_, *A*_1_) and *H*(*v*_2_, *A*_2_ − ∇*f*), which leads to

$$\begin{array}{c} \left[H\left(v_{1},A_{1}\right)-H(v_{2},A_{2}-\nabla f)\right]\psi_{2}\;=\sum_{j=1}^{N}\left(v_{1}\left(x_{j}\right)-v_{2}\left(x_{j}\right)\right)\psi_{2}\;=\left(V_{1}-V_{2}\right)\psi_{2}=\left(e_{1}-e_{2}\right)\psi_{2}. \end{array}$$

However, Theorem 6 allows us to conclude *ψ*_2_ ≠ 0 and it follows *v_1_* = *v*_2_ + constant.

Theorem 10 *Assume* ∇ × A_1_ = ∇ × A_2_ = B *and that* v_k_, A_k_ *for* k = 1,2, *and* B *fulfill Assumption 1 and take the requirements of Theorem* 6 *for* H(v_1_,A_1_) *and* H(v_2_,A_2_) *to hold. If the ground‐state particle densities satisfy* ρ_1_ = ρ_2_, *then* v_1_ = v_2_ + C *almost everywhere for some constant* C.

Remark 7 Theorem 10 is the Hohenberg‐Kohn theorem for BDFT, first established by Grayce and Harris^33^ but missing the UCP argument (see also Reference 9).

Theorem 10 can be used to obtain

Corollary 11 *Assume Assumption 1 and the requirements of Theorem 6 for* H(v_1_,A_1_) *and* H(v_2_,A_2_) *and that the ground‐state densities fulfill* ρ = ρ_1_ = ρ_2_, j = j_1_ = j_2_. *For systems with* j^p^ = 0, *it follows* B_1_ = B_2_ *(even* A_1_ = A_2_ *holds) and* v_1_ = v_2_ + C *for some constant* C.

*Proof*. For systems with *j^p^* = 0, *j*_1_ = *j*_2_ implies

$$\rho A_{1}=\rho A_{2},$$

since *ρ*_1_ = *ρ*_2_ = *ρ*. Theorem 6 gives *ρ* > 0 almost everywhere and we may conclude *A*_1_ = *A*_2_. Theorem 10 now gives the equality *v*_1_ = *v*_2_ + *C* for some constant *C*. □

## 6 PROOFS OF THE MAIN RESULTS

Proof of Theorem 6 *In the sequel let* D = 3 N. *By Assumption* 1, *the strong UCP holds for Equation* (3) *by Theorem* 4. *Next, we follow the proof of Theorem 1.2 given after Lemma 3.3 in Regbaoui* 26 *and Lemma A.2 in Lammert*.^30^ *(Lemma A.2 corresponds to setting* A = 0 *here, and moreover we exploit* u ≥ 0 *instead of the assumption* u(x_1_,x_2_) = u^′^(x_1_ − x_2_).) *We start by showing the following inverse Poincaré inequality for solutions of the Schrödinger equation*:

For an arbitrary point $x_{0}={\left(x_{0;j}\right)}_{j=1}^{N}\;\in \;{\mathbb{R}}^{D}$ and *r* ≤ *r*_0_

(13) $$\int_{B_{r}\left(x_{0}\right)}{\left|\nabla \psi \left(x\right)\right|}^{2}dx\leq \frac{C}{r^{2}}\int_{B_{2r}\left(x_{0}\right)}{\left|\psi \left(x\right)\right|}^{2}dx.$$

Here *C* is a positive constant that depends on *r*_0_ > 0, *v*, and *A* (but is independent of *u* ≥ 0).

Choose $h\;\in \;C_{0}^{\infty }\left(B_{2r}\left(x_{0}\right)\right)$ that satisfies *h*(*x*) = 1 if |*x* − *x*_0_| ≤ *r*, *h* ≤ 1 for |*x* − *x*_0_| ≤ 2*r*, and |∇*h*(*x*)| ≤ 2*r* ^−1^. In the Schrödinger equation (4), we choose *φ* = *h* ^2^ *ψ* and move all terms except one to the right hand side so that

(14) $$\begin{array}{c} \int_{{\mathbb{R}}^{D}}{\left|h\nabla \psi \right|}^{2}dx=-2\int_{{\mathbb{R}}^{D}}h\left(\nabla \psi \right)\cdot \bar{\psi }\nabla h\;dx\;-2i\int_{{\mathbb{R}}^{D}}\underline{A}\cdot \left(\nabla \psi \right)h^{2}\bar{\psi }\;dx+\int_{{\mathbb{R}}^{D}}\left(e-W_{A}\right){\left|\mathrm{h\psi}\right|}^{2}dx. \end{array}$$

We now bound each of the terms of the right hand side in Equation (14).

It is immediate that the first term is less or equal to 2∥*h*∇*ψ*∥_2_∥*ψ*∇*h*∥_2_. Using the inequality 2*ab* ≤ *a* ^2^/6 + 6*b* ^2^, we obtain an upper bound

(15) $$\frac{1}{6}\int_{{\mathbb{R}}^{D}}{\left|h\nabla \psi \right|}^{2}dx+6{\left\|\psi \nabla h\right\|}_{2}^{2}.$$

To continue, let $I_{1}=-2i\int_{{\mathbb{R}}^{D}}\underline{A}\cdot \left(\nabla \psi \right)h^{2}\bar{\psi }\;dx$. The Cauchy‐Schwarz inequality together with 2*ab* ≤ *a* ^2^/6 + 6*b* ^2^ yield

(16) $$I_{1}\leq \frac{1}{6}\int_{{\mathbb{R}}^{D}}{\left|h\nabla \psi \right|}^{2}dx+6\int_{{\mathbb{R}}^{D}}{\left|\underline{A}\right|}^{2}{\left|\mathrm{h\psi}\right|}^{2}dx.$$

For the last term of the right hand side in Equation (14), we use the definition of *W*_*A*_ and write $e-W_{A}=e-W-{\left|\underline{A}\right|}^{2}-i\left(\nabla \cdot \underline{A}\right)$. Thus

$$\begin{array}{c} \int_{{\mathbb{R}}^{D}}\left(e-W_{A}\right){\left|\mathrm{h\psi}\right|}^{2}dx=\int_{{\mathbb{R}}^{D}}\left(e-W-{\left|\underline{A}\right|}^{2}\right){\left|\mathrm{h\psi}\right|}^{2}dx\;-i\int_{{\mathbb{R}}^{D}}\left(\nabla \cdot \underline{A}\right){\left|\mathrm{h\psi}\right|}^{2}dx \end{array}$$

and it follows from *W* = *V*_+_ + *U*_+_ − *V*_−_ ≥ −*V*_−_ that

(17) $$\begin{array}{c} \int_{{\mathbb{R}}^{D}}\left(e-W_{A}\right){\left|\mathrm{h\psi}\right|}^{2}dx\leq \int_{{\mathbb{R}}^{D}}\left(V_{-}+,|,e,|\right){\left|\mathrm{h\psi}\right|}^{2}dx\;+\int_{{\mathbb{R}}^{D}}{\left|\left(\nabla \cdot \underline{A}\right)\|\mathrm{h\psi}\right|}^{2}dx. \end{array}$$

Define $\Theta =V_{-}+|e|+6{\left|\underline{A}\right|}^{2}+|\nabla \cdot \underline{A}|$, from Equations 15, 16, 17 we obtain an upper bound for the right hand side of Equation (14) given by

(18) $$\frac{1}{3}\int_{{\mathbb{R}}^{D}}{\left|h\nabla \psi \right|}^{2}dx+6{\left\|\psi \nabla h\right\|}_{2}^{2}+\int_{{\mathbb{R}}^{D}}\Theta {\left|\mathrm{h\psi}\right|}^{2}dx.$$

With the notation Θ_1_ = *v*_−_ + *N* ^−1^ ∣ *e* ∣  + 6|*A*|^2^ +  ∣∇ ⋅ *A*∣, the inequality $\Theta \left(x\right)\leq \sum_{j=1}^{N}\Theta_{1}\left(x_{j}\right)$ holds. Furthermore, we have

$$\int_{{\mathbb{R}}^{D}}\Theta \left(x\right){\left|\mathrm{h\psi}\right|}^{2}dx\leq \sum_{j=1}^{N}\int_{{\mathbb{R}}^{D}}\Theta_{1}\left(x_{j}\right){\left|\mathrm{h\psi}\right|}^{2}dx=I_{2},$$

where the last equality defines *I*_2_.

By assumption $v_{-}\;\in \;L_{\mathrm{loc}}^{3/2}\left({\mathbb{R}}^{3}\right)$, ${\left|A\right|}^{2}\;\in \;L_{\mathrm{loc}}^{2}\left({\mathbb{R}}^{3}\right)$, and $\nabla \cdot A\;\in \;L_{\mathrm{loc}}^{2}\left({\mathbb{R}}^{3}\right)$, and it follows that $\Theta_{1}\;\in \;L_{\mathrm{loc}}^{3/2}\left({\mathbb{R}}^{3}\right)$. To bound the term *I*_2_ from above, we closely follow Lammert^30^ and define $\tilde{\rho }\left(x_{1}\right)=N\int_{{\mathbb{R}}^{3\left(N-1\right)}}{\left|\mathrm{h\psi}\right|}^{2}{dx}_{2}\cdots {dx}_{N}$. For *M* > 0 we let $M\prime ={\left\|\Theta_{1}\chi_{B_{2r}(x_{0;1})}\chi_{\left\{\Theta_{1}\geq M\right\}}\right\|}_{3/2}$, where the characteristic function of a set *X* is denoted *χ_X_*. Hölder's inequality gives

$$\begin{array}{cc} I_{2}=\int_{\left\{\Theta_{1}<M\right\}}\Theta_{1}\tilde{\rho }\;{dx}_{1}+\int_{\left\{\Theta_{1}\geq M\right\}}\Theta_{1}\tilde{\rho }\;{dx}_{1} & \leq M{\left\|\mathrm{h\psi}\right\|}_{2}^{2}+M^{\prime }{\left\|\tilde{\rho }\right\|}_{3}, \end{array}$$

and by a Sobolev inequality ${\left\|\tilde{\rho }\right\|}_{3}\leq C{\left\|\nabla \left({\tilde{\rho }}^{1/2}\right)\right\|}_{2}^{2}$. A direct computation of ∇$({\tilde{\rho }}^{1/2}),$ using the definition of $\tilde{\rho },$ shows that ${\left\|\nabla \left({\tilde{\rho }}^{1/2}\right)\right\|}_{2}^{2}\leq \int_{{\mathbb{R}}^{D}}{\left|\nabla \left(\mathrm{h\psi}\right)\right|}^{2}dx$ (see also the original argument of Lieb,^31^ Theorem 1.1). From |*a* + *b*|^2^ ≤ 2|*a*|^2^ + 2|*b*|^2^, we get

$${\left\|\nabla \left({\tilde{\rho }}^{1/2}\right)\right\|}_{2}^{2}\leq 2\int_{{\mathbb{R}}^{D}}{\left|h\nabla \psi \right|}^{2}dx+2{\left\|\psi \nabla h\right\|}_{2}^{2}.$$

We choose *M* > 0 such that 2*CM* ^*′*^ ≤ 1/6 and then one has

(19) $$I_{2}\leq \frac{1}{6}\int_{{\mathbb{R}}^{D}}{\left|h\nabla \psi \right|}^{2}dx+\frac{1}{6}{\left\|\psi \nabla h\right\|}_{2}^{2}+M{\left\|\mathrm{h\psi}\right\|}_{2}^{2}.$$

Returning to Equation (18), we set $C=\left[74+3{\mathrm{Mr}}_{0}^{2}\right]/3$ and use Equation (19) and |∇*h*| ≤ 2/*r* to conclude for *r* ≤ *r*_0_

$$\begin{array}{c} \frac{1}{2}\int_{{\mathbb{R}}^{D}}{\left|h\nabla \psi \right|}^{2}dx\leq \frac{37}{6}{\left\|\psi \nabla h\right\|}_{2}^{2}+M{\left\|\mathrm{h\psi}\right\|}_{2}^{2}\leq \frac{C}{r^{2}}\int_{B_{2r}\left(x_{0}\right)}{\left|\psi \right|}^{2}dx. \end{array}$$

Hence Equation (13) holds.

Suppose $\psi \;\in \;H_{\mathrm{loc}}^{2}$ vanishes on a set *E* of positive measure. Almost every point of *E* is a density point. Let *x*_0_ be such a density point and let *B*_*r*_ = *B*_*r*_(*x*_0_). Given *ε* > 0 there is an *r*_0_ = *r*_0_(*ε*) so that (cf. (3.11) in Regbaoui^26^)

(20) $$\frac{|E\cap B_{r}|}{|B_{r}|}\geq 1-\varepsilon ,\;\frac{|E^{c}\cap B_{r}|}{|B_{r}|}\leq \varepsilon ,\;\text{for}\;r\leq r_{0}.$$

Lemma 3.3 in Regbaoui^26^ (or Lemma 3.4 in Ladyzenskaya‐Ural'tzeva^34^) gives

(21) $$\int_{B_{r}\cap E^{c}}{\left|\psi \right|}^{2}dx\leq C\frac{r^{D}}{|E|}{\left|B_{r}\cap E^{c}\right|}^{1/D}\int_{B_{r}}|\nabla \left(\psi^{2}\right)|dx$$

for some constant *C*. Applying the Cauchy‐Schwarz inequality to the right hand side of Equation (21), we obtain

$$\int_{B_{r}}{\left|\psi \right|}^{2}dx\leq C\frac{r^{2D}}{{\left|E\right|}^{2}}{\left|B_{r}\cap E^{c}\right|}^{2/D}\int_{B_{r}}{\left|\nabla \psi \right|}^{2}dx$$

for some new constant *C*. Since |*E*| ≥ |*E* ∩ *B*_*r*_|, Equation (20), and the inverse Poincaré inequality (13) allow us to conclude that

(22) $$\begin{array}{c} \int_{B_{r}}{\left|\psi \right|}^{2}dx\leq C\frac{\varepsilon^{2/D}}{{\left(1-\varepsilon \right)}^{2}}r^{2}\int_{B_{r}}{\left|\nabla \psi \right|}^{2}dx\leq C^{\prime }\frac{\varepsilon^{2/D}}{{\left(1-\varepsilon \right)}^{2}}\int_{B_{2r}}{\left|\psi \right|}^{2}dx. \end{array}$$

Introduce the function $f\left(r\right)=\int_{B_{r}}{\left|\psi \right|}^{2}dx$, fix an integer *n* and choose *ε* > 0 so that *C* ^*′*^ *ε* ^2/*D*^/(1 − *ε*)^2^ = 2^−*n*^. Then Equation (22) can be written *f*(*r*) ≤ 2^−*n*^ *f*(2*r*). By iteration

$$f\left(r^{\prime }\right)\leq 2^{-\mathrm{kn}}f\left(2^{k}r^{\prime }\right),\;r^{\prime }\leq 2^{1-k}r_{0}$$

holds. For fixed *r* and *k* chosen such that 2^−*k*^ *r*_0_ ≤ *r* ≤ 2^1 − *k*^ *r*_0_ , it follows that

$$f\left(r\right)\leq 2^{-\mathrm{kn}}f\left(2r_{0}\right)\leq {\left(\frac{r}{r_{0}}\right)}^{n}f\left(2r_{0}\right),$$

where *r*_0_ depends on *n*. Consequently *f* vanishes to infinite order, that is, for all *m* there is an *r*_0_(*m*) such that

$$f\left(r\right)=\int_{B_{r}}{\left|\psi \right|}^{2}dx\leq C_{m}r^{m},\;r\leq r_{0}\left(m\right).$$

That *ψ* = 0 follows now by the strong UCP given by Theorem 4. □

Proof of Corollary 1 *This is a consequence of the proof of Theorem 6, since Θ_1_, by assumption, is an element of* $L_{\mathrm{loc}}^{3/2}\left({\mathbb{R}}^{3}\right)$. □

Proof of Corollary 2 *We first demonstrate that the conditions of Corollary* 8 *fulfills Assumption* 1. *Due to the particular form of the potentials, we make use of the following: Let* $f_{1}\;\in \;K_{\mathrm{loc}}^{3,\delta }$ *and* $f_{2}\;\in \;K_{\mathrm{loc}}^{6,\delta }$. *Then both* $\sum_{k=1}^{N}f_{1}\left(x_{k}\right)$ *and* ∑_*k* ≠ *l*_ *f*_2_(*x*_*k*_, *x*_*l*_) *are elements of* $K_{\mathrm{loc}}^{3N,\delta }$. *Similar statements for* K^n^ *can be found in Simon* 35 *(Example F) and Aizenman‐Simon* 36 *(Theorem 1.4). We prove our claim by direct computations. Define* $I_{1}^{\delta }$ *and* $I_{2}^{\delta }$ *according to*

$$\begin{array}{l} I_{1}^{\delta }\left(x\right)=\sum_{j=1}^{N}\int_{B_{r}\left(0\right)}\frac{|f_{1}\left(y_{j}+x_{j}\right)|}{{\left(y_{1}^{2}+\cdots +y_{N}^{2}\right)}^{\frac{3N-2+\delta }{2}}}{dy}_{1}\cdots {dy}_{N},\\ I_{2}^{\delta }\left(x\right)=\sum_{j\neq l}\int_{B_{r}\left(0\right)}\frac{|f_{2}\left(y_{j}+x_{j},y_{l}+x_{l}\right)|}{{\left(y_{1}^{2}+\cdots +y_{N}^{2}\right)}^{\frac{3N-2+\delta }{2}}}{dy}_{1}\cdots {dy}_{N}. \end{array}$$

We next demonstrate that

(23) $$I_{1}^{\delta }\left(x\right)\leq C_{N}\int_{B_{r;3}\left(x\right)}\frac{|f_{1}\left(y_{1}\right)|}{{\left|y_{1}-x_{1}\right|}^{3-2+\delta }}{dy}_{1},$$

(24) $$I_{2}^{\delta }\left(x\right)\leq C_{N}\int_{B_{r;6}\left(x\right)}\frac{|f_{2}\left(y_{1},y_{2}\right)|}{{\left|\left(y_{1},y_{2}\right)-(x_{1},x_{2})\right|}^{6-2+\delta }}{dy}_{1}{dy}_{2},$$

where the second index in the given ball‐sets *B*_*r*; 3_(*x*) ⊂ ℝ^3^ and *B*_*r*; 6_(*x*) ⊂ ℝ^6^ refers to the respective dimensionality.

To show Equation (23), set *q* = (*y*_2_, …, *y*_*N*_) and note that

$$\begin{array}{l} I_{1}^{\delta }\left(x\right)\leq N\int_{B_{r;3}\times B_{r;3\left(N-1\right)}}\frac{|f_{1}\left(y_{1}+x_{1}\right)|}{{\left(y_{1}^{2}+q^{2}\right)}^{\frac{3N-2+\delta }{2}}}{dy}_{1}dq=C_{N}\int_{B_{r;3}}|f_{1}\left(y_{1}+x_{1}\right)|\left(\int_{0}^{r}\frac{q^{3\left(N-1\right)-1}dq}{{\left(y_{1}^{2}+q^{2}\right)}^{\frac{3N-2+\delta }{2}}}\right){dy}_{1}\\ =C_{N}\int_{B_{r;3}}\frac{|f_{1}\left(y_{1}+x_{1}\right)|}{{\left|y_{1}\right|}^{3N-2+\delta }}\;\left(\int_{0}^{r}\frac{q^{3N-4}dq}{{\left(1+{\left(q/,|,y_{1},|\right)}^{2}\right)}^{\frac{3N-2+\delta }{2}}}\right){dy}_{1}\\ \leq C_{N}\;J_{1}^{\delta }\int_{B_{r;3}\left(x_{1}\right)}\frac{|f_{1}\left(y_{1}\right)|}{{\left|y_{1}-x_{1}\right|}^{3-2+\delta }}{dy}_{1}, \end{array}$$

where we have defined the integral

$$J_{1}^{\delta }=\int_{0}^{\infty }\frac{s^{3N-4}}{{\left(1+s^{2}\right)}^{\frac{3N-2+\delta }{2}}}ds.$$

Now, $J_{1}^{\delta }$ is finite since

$$J_{1}^{\delta }\leq \int_{0}^{1}s^{3N-4}ds+\int_{1}^{\infty }s^{-2-\delta }ds<+\infty .$$

This establishes Equation (23). The proof of Equation (24) is similar and included for the sake of completeness. Set *q* = (*y*_3_,…,*y*_*N*_), then

$$\begin{array}{l} I_{1}\leq N\int_{B_{r;6}\times B_{r;3\left(N-2\right)}}\frac{|u\left(y_{1}+x_{1},y_{2}+x_{2}\right)|}{{\left(y_{1}^{2}+y_{2}^{2}+q^{2}\right)}^{\frac{3N-2+\delta }{2}}}{dy}_{1}{dy}_{2}dq=C_{N}\int_{B_{r;6}}|u\left(y_{1}+x_{1},y_{2}+x_{2}\right)|\;\left(\int_{0}^{r}\frac{q^{3\left(N-2\right)-1}dq}{{\left(y_{1}^{2}+y_{2}^{2}+q^{2}\right)}^{\frac{3N-2+\delta }{2}}}\right){dy}_{1}{dy}_{2}\\ =C_{N}\int_{B_{r;6}}\frac{|u\left(y_{1}+x_{1},y_{2}+x_{2}\right)|}{{\left|\left(y_{1},y_{2}\right)\right|}^{3N-2+\delta }}\;\left(\int_{0}^{r}\frac{q^{3N-7}dq}{{\left(1+{\left(q/,|,\left(y_{1},y_{2}\right),|\right)}^{2}\right)}^{\frac{3N-2+\delta }{2}}}\right){dy}_{1}{dy}_{2}\\ \leq C_{N}\int_{B_{r;6}\left(x_{1},x_{2}\right)}\frac{|u\left(y_{1},y_{2}\right)|}{{\left|\left(y_{1},y_{2}\right)-(x_{1},x_{2})\right|}^{6-2+\delta }}{dy}_{1}{dy}_{2}\;\int_{0}^{\infty }\frac{s^{3N-7}}{{\left(1+s^{2}\right)}^{\frac{3N-2+\delta }{2}}}ds. \end{array}$$

Corollary 8 now follows from Theorem 6 (Equation (5) in Assumption 1 is fulfilled by Remark 3). □

Proof of Corollary 3 *We first reduce the molecular case to the atomic one. Since the UCP from sets of positive measure is local, it can be applied to any open set in the domain individually. So instead of one singularity (the* y *of Assumption* 1 *), we can treat an arbitrary (yet countable) number of singularities if they do not have an accumulation point. For this just choose an open cover* {*U*_*j*_} *of* ℝ^3^ *where each U*_*j*_ *contains not more than one nucleus* *x*_nuc; *j*_ . *It remains to show that all* $q_{x_{\mathrm{nuc};j}},\;b_{x_{\mathrm{nuc};j}}$ *belong to the respective local Kato classes and we are done if we prove the results for atoms*.

In the sequel we let *v*(*x*_1_) =  − *Z*|*x*_1_ − *x*_nuc_|^−1^, *x*_nuc_ ∈ ℝ^3^ , *Z* > 0, and *u*(*x*_1_, *x*_2_) = |*x*_1_ − *x*_2_|^−1^ . In this case $v_{-}\;\in \;L_{\mathrm{loc}}^{3/2}\left({\mathbb{R}}^{3}\right)$ and with the choice ${\underline{x}}_{\mathrm{nuc}}=\left(x_{\mathrm{nuc}},\ldots ,x_{\mathrm{nuc}}\right)\;\in \;{\mathbb{R}}^{D}$, we have with $Q_{x_{\mathrm{nuc}}}\left(x\right)=Q_{{\underline{x}}_{\mathrm{nuc}}}\left(x\right)$ the equality

$$\begin{array}{l} Q_{x_{\mathrm{nuc}}}\left(x\right)=(\sum_{j}\frac{-2Z}{|x_{j}-x_{\mathrm{nuc}}|}+\sum_{j\neq l}\frac{1}{|x_{j}-x_{l}|}\;+\sum_{j}\left(x_{j}-x_{\mathrm{nuc}}\right)\cdot \nabla_{j}\left(\frac{-Z}{|x_{j}-x_{\mathrm{nuc}}|}\right)\;+\frac{1}{2}\sum_{j\neq l}\left[\left(x_{j}-x_{\mathrm{nuc}}\right)\cdot \nabla_{j}+\left(x_{l}-x_{\mathrm{nuc}}\right)\cdot \nabla_{l}\right]\;\frac{1}{|\left(x_{j}-x_{\mathrm{nuc}}\right)-\left(x_{l}-x_{\mathrm{nuc}}\right)|}){\;}_{-}\\ ={\left(V\left(x\right)+U\left(x\right)\right)}_{-}\leq 2V_{-}\left(x\right)\;. \end{array}$$

Thus, in this case we can choose $q_{1,x_{\mathrm{nuc}}}=v_{-}$ and $q_{2,x_{\mathrm{nuc}}}=0$.

Furthermore, for 0 < *δ* < 1, we claim that $V,U\;\in \;K_{\mathrm{loc}}^{D,\delta }$. By the first part it suffices to show $v\;\in \;K_{\mathrm{loc}}^{3,\delta }$ and $u\;\in \;K_{\mathrm{loc}}^{6,\delta }$. For $v\;\in \;K_{\mathrm{loc}}^{3,\delta }$, we introduce polar coordinates with radius *s* and polar angle *t*. Then it holds that *dy* = 2*πs* ^2^sin*t* d*t*d*s* and *y* · *x* = −*s*|*x*|*cost*. For *f*_1_(*x*) = |*x*|^−1^ it follows that

$$\eta^{K}\left(r;f_{1}\right)=\underset{|x|\leq R}{\sup}\int_{0}^{r}\left(\int_{0}^{\pi }\frac{2\pi s^{1-\delta }\sin\;t\;dt}{{\left(s^{2}-2s|x|\cos\;t+{\left|x\right|}^{2}\right)}^{1/2}}\right)ds.$$

We integrate over *t*, use |*s* + |*x*| − |*s* − |*x*|||≤2|*x*|, and the conclusion is obtained for *v*. In a similar fashion, for *u* we establish that with *f*_2_(*x*) = |*x*_1_ − *x*_2_|^−1^

$$\eta^{K}\left(r;f_{2}\right)\leq C\int_{0}^{r}\left(\int_{s_{1}}^{r}\frac{s_{2}\;{ds}_{2}}{{\left(s_{1}^{2}+s_{2}^{2}\right)}^{2+\frac{2}{\delta }}}\right)s_{1}^{2}{ds}_{1}\leq {\mathrm{Cr}}^{1-\delta }$$

such that it follows $u\;\in \;K_{\mathrm{loc}}^{6,\delta }$, since

$$\begin{array}{l} \int_{B_{r}\left(0\right)}\frac{1}{|y_{1}-y_{2}|}\frac{1}{{\left|y\right|}^{6-2+\delta }}{dy}_{1}{dy}_{2}\leq C\int_{0}^{r}\int_{0}^{r}\left(\int_{0}^{\pi }\frac{2\pi s_{1}^{2}s_{2}^{2}\;\sin\;t\;dt}{{\left(s_{1}^{2}-2s_{1}s_{2}\;\cos\;t+s_{2}^{2}\right)}^{1/2}}\right)\;\frac{{ds}_{1}{ds}_{2}}{{\left(s_{1}^{2}+s_{2}^{2}\right)}^{2+\frac{2}{\delta }}}\\ \leq C\int_{0}^{r}\int_{0}^{r}\frac{\left(s_{1}+s_{2}-,|,s_{1}-s_{2},|\right)}{{\left(s_{1}^{2}+s_{2}^{2}\right)}^{2+\frac{2}{\delta }}}s_{1}s_{2}{ds}_{1}{ds}_{2}\\ \leq C\int_{0}^{r}\left[\int_{0}^{s_{1}}\frac{s_{1}s_{2}^{2}{ds}_{2}}{{\left(s_{1}^{2}+s_{2}^{2}\right)}^{2+\frac{2}{\delta }}}+\int_{s_{1}}^{r}\frac{s_{1}^{2}s_{2}{ds}_{2}}{{\left(s_{1}^{2}+s_{2}^{2}\right)}^{2+\frac{2}{\delta }}}\right]{ds}_{1}\\ \leq C\int_{0}^{r}\int_{s_{1}}^{r}\frac{s_{2}{ds}_{2}}{{\left(s_{1}^{2}+s_{2}^{2}\right)}^{2+\frac{2}{\delta }}}s_{1}^{2}{ds}_{1}\leq {\mathrm{Cr}}^{1-\delta }. \end{array}$$

The atomic case is now a consequence of Corollary 8. □

## 7 CONCLUSION

In this work we were able to show the unique‐continuation property from sets of positive measures for the important case of the many‐body magnetic Schrödinger equation for classes of potentials that are independent of the particle number. This is crucial in order to not artificially restrict the permitted potentials in large systems. We further specifically addressed molecular Hamiltonians, thus covering most cases that usually arise in physics.
